# Prevalence of Thyroid Autoimmunity in Children with Celiac Disease Compared to Healthy 12-Year Olds

**DOI:** 10.1155/2014/417356

**Published:** 2014-01-27

**Authors:** Maria van der Pals, Anneli Ivarsson, Fredrik Norström, Lotta Högberg, Johan Svensson, Annelie Carlsson

**Affiliations:** ^1^Department of Pediatrics, Clinical Sciences, Skåne University Hospital, Lund University, 205 02 Lund, Sweden; ^2^Department of Public Health and Clinical Medicine, Epidemiology and Global Health, Umeå University, 905 85 Umeå, Sweden; ^3^Division of Pediatrics, Department of Clinical and Experimental Medicine, Faculty of Health Sciences, Linköping University, 581 85 Linköping, Sweden; ^4^Department of Pediatrics in Norrköping, County Council of Östergötland, 603 79 Norrköping, Sweden

## Abstract

*Objectives*. Studies have suggested a correlation between untreated celiac disease and risk for other autoimmune diseases. We investigated the prevalence of thyroid autoimmunity in 12-year-old children (i) with symptomatic celiac disease diagnosed and treated with a gluten-free diet, (ii) with screening-detected untreated celiac disease, and (iii) without celiac disease. *Methods*. Blood samples from 12632 children were collected. All celiac disease cases, previously diagnosed and newly screening-detected, were identified. Per case, 4 referents were matched. Blood samples were analyzed for autoantibodies against thyroid peroxidase (TPOAb). The cut-off value for TPO positivity was set to 100 U/mL. *Results*. Altogether, 335 celiac disease cases were found. In the entire celiac disease group, 7.2% (24/335) had elevated titers of TPOAb compared to 2.8% (48/1695) of the referents. Among the previously diagnosed celiac disease cases, 7.5% (7/93, OR 2.8, 95% CI 1.2–6.4) was TPOAb positive and among screening-detected cases, 7.0% (17/242, OR 2.6, 95% CI 1.5–4.6) was TPOAb positive. *Conclusion*. Children with celiac disease showed a higher prevalence of thyroid autoimmunity. We could not confirm the hypothesis that untreated celiac disease is associated with increased risk of developing thyroid autoimmunity. Early initiation of celiac disease treatment might not lower the risk for other autoimmune diseases.

## 1. Introduction 

Celiac disease is one of the most common chronic diseases in childhood, affecting approximately 0.5–3% of the population in the Western world [1–3]. It is characterized by an autoimmune response triggered by gluten and possibly other environmental cofactors, leading to small-intestinal mucosal injury [4]. This in turn leads to malabsorption with a variable clinical expression ranging from no symptoms to severe malnutrition. The disease can have its onset at any age throughout life [5, 6]. The human leukocyte antigen HLA DQ2 or DQ8 haplotype is carried by all celiac disease cases, and the prevalence in the general population has been assumed to be about 30% [7, 8]. The presence of HLA DQ2 and/or DQ8 is necessary but not sufficient for development of the disease. Studies of identical twins and siblings suggest the HLA contribution to be less than 50%, with the remaining part being explained by a combination of non-HLA genes and environmental factors [9].

Autoimmune conditions including thyroid diseases such as Hashimoto thyroiditis and Grave's disease are associated with celiac disease [10–13] with a reported prevalence up to 10 times that in the general population [14–17]. Some studies have suggested that untreated celiac disease and thus also gluten exposure, with subsequent inflammation and mucosal injury, increase the risk for developing other autoimmune diseases such as thyroid diseases and insulin dependent diabetes mellitus. Ventura et al. showed that patients with celiac disease had a high prevalence of both insulin dependent diabetes mellitus autoantibodies and thyroid-related serum autoantibodies. Moreover, these autoantibodies were supposed to be gluten-dependent, because they disappeared during treatment with a gluten-free diet [12, 18].

These findings raise questions as to whether abnormal immune responses, at the level of the gut mucosa when exposed to environmental antigens, play a role in systemic autoimmune disease or if these associations instead reflect an underlying joint genetic predisposition. If the duration of gluten exposure is positively correlated to development of autoimmune disease, and if early detection and initiated treatment of the disease reduces the risk for development of autoimmune disease, this would point in the direction of a general celiac disease screening at as early an age as possible.

The aim of our study was to investigate the prevalence of thyroid autoimmunity in 12-year-old children with celiac disease compared to sex-matched referents and to investigate if early introduction of a gluten-free diet in children with celiac disease reduces the risk for developing autoimmune thyroid disease. The hypothesis is that previously diagnosed cases with a consequentially reduced exposure (after the time of the diagnosis) can be expected to display a lower incidence of TPOAbs than screening-detected cases (with ongoing exposure).

## 2. Materials and Methods

### 2.1. A Population-Based Celiac Disease Screening Study

A two-phased population-based cross-sectional multicenter screening study for celiac disease in 12-year olds was performed in 2005-2006 and 2009-2010 representing two birth cohorts (children born in 1993 and 1997, resp.) [3, 19]. The study was entitled the ETICS study (Exploring the Iceberg of Celiacs in Sweden) and was part of the PreventCD European project [3, 20]. Both screening efforts covered the same geographical areas and followed a similar protocol, including collaboration with school health services. Families gave their signed informed consent before being enrolled. The study was approved by the Regional Ethical Review Board of Umeå University, Umeå, Sweden.

In total, 12632 children (69% of those invited) participated, with similar sex ratios in both birth cohorts (48% and 49% girls in the 1993 and 1997 groups, resp.). Details of the celiac disease screening strategy and descriptions of both cohorts have been published previously [3, 19]. In brief, blood samples from all participating children were analyzed for antihuman tissue transglutaminase and if borderline values were obtained also for endomysial antibodies (both of isotype IgA). Children with values above a predefined cut-off were referred to the closest pediatric clinic for a small intestinal biopsy, which represents the gold standard for diagnosis [21, 22]. Criteria for diagnosis were Marsh 3a–c enteropathy or the combination of Marsh 1-2 enteropathy, HLA-DQ2/DQ8 haplotype, and symptoms and/or signs compatible with celiac disease [22].Genotyping for HLA alleles encoding for HLA-DQ2/DQ8 was performed by oligonucleotide probe hybridization and was verified in all screening-detected cases. For those who reported clinically detected celiac disease, diagnosis was confirmed by review of histology and serological markers from the National Swedish Childhood Celiac Disease Register [23] and/or medical records.

### 2.2. A Nested Case-Referent Study on Thyroid Autoimmunity

A case-referent design nested within the ETICS study was used to evaluate the risk of thyroid autoimmunity related to treated (i.e., the previously diagnosed celiac disease cases) and untreated celiac disease, respectively. The mean age at diagnosis among the previously diagnosed celiac disease cases was 4.7 and the median was 2.75 years. They had thus been on a gluten-free diet for approximately 7 to 9 years since the screening was performed at 12 years of age. The total number of celiac disease cases from both screening efforts, including previously diagnosed cases and newly screening-detected cases, together formed our celiac disease case group. This cohort was used as a basis for screening of thyroid autoimmunity, defined as significant titers of TPOAb. The blood samples drawn for the TPOAbs were taken at 12 years of age, that is, at the time of the screening. Autoantibodies of IgG type directed against thyroid peroxidase (TPOAb) that were measured in blood samples were used as an indicator of thyroid autoimmunity and expressed as arbitrary units per milliliter (U/mL). The cut-off value for TPO positivity was set to 100 U/mL (Varelisa TPO Antibodies, Phadia GmbH, Freiburg, Germany).

Four referents were selected for each celiac disease case. Referents, matched for sex, were randomly selected from all cohort members free of celiac disease at the time of diagnosis. In this paper we included cases with a confirmed celiac disease diagnosis, obtained either through the ETICS screening (*n* = 242) or ahead of the screening (*n* = 93). We therefore excluded 90 children, either because of incorrect information about the existing celiac disease diagnosis (*n* = 36 children) or because of no biopsy verifying a celiac disease diagnosis (*n* = 54 children). This resulted in 213 cases and 1150 referents from the first phase (a rate of 5.4 referents per case) and 122 cases and 545 referents from the second phase (a rate of 4.5 referents per case) for the analyses in this paper.

### 2.3. Statistical Analysis

The relation between celiac disease and TPOAb positivity was analyzed with logistic regression using the nonceliac disease children as the reference group. Results are presented as odds ratios (OR) with 95% confidence intervals (CI). Microsoft Access was used for data handling and Stata 10 for statistical analysis (StataCorp LP, College Station, TX). Statistical significance was accepted at *P* < 0.05 corresponding to a CI not including 1.

## 3. Results

### 3.1. Study Population Characteristics

**Figure 1:**
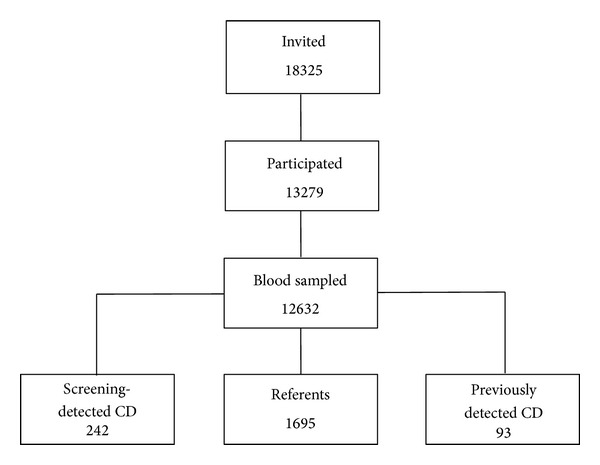
Flowchart depicting the screening procedure. Cross-sectional screenings performed in 12-year olds across Sweden to investigate the total prevalence of celiac disease (CD), including both clinically- and screening-detected cases. Numbers of children are given in the boxes.

In total, among the 12632 children, we identified 335 celiac disease cases whereof 93 had previously diagnosed celiac disease and 242 were detected within the study (Figure 1). Details regarding the prevalence of celiac disease have been published elsewhere, but in short, the screening procedure revealed a total celiac disease prevalence of 29/1 000 in the 1993 cohort, including both previously and screening-detected cases, and 22/1 000 in the 1997 cohort [3, 19]. The proportions of children with previously diagnosed celiac disease and those with screening-detected disease were similar in the cohorts. The mean age at diagnosis among the previously diagnosed children was 4.7 years (SD 4.0) and the median was 2.75 years.

### 3.2. Thyroid Autoimmunity

In the celiac disease group, 7.2% (24/335) had elevated titers of TPOAb compared to 2.8% (48/1695) of the referents. Among the previously diagnosed celiac disease cases 7.5% (7/93, OR 2.8, 95% CI 1.2–6.4) was TPOAb positive and among the screening-detected cases 7.0% (17/242, OR 2.6, 95% CI 1.5–4.6) was TPOAb positive (Table 1).

## 4. Discussion

This study demonstrates an increased prevalence of thyroid autoimmunity among 12-year-old children with celiac disease compared to healthy controls. We found the prevalence of thyroid autoimmunity to be almost three times higher than in the age- and sex-matched control group of children without celiac disease. We did not find any difference in the prevalence of thyroid autoantibodies in the group of children with previously diagnosed celiac disease compared to the screening-detected cases. Our previous pilot screening studies, performed on the same birth cohorts, showed that 2.5-year olds have approximately the same prevalence of undiagnosed celiac disease as found in the ETICS study [24]. This supports the theory that not all cases detected through screening are of recent onset and these may constitute a group of children who have had untreated celiac disease since early childhood [24, 25]. The previously diagnosed cases had already been treated for several years with a gluten-free diet. The mean age at diagnosis among the previously diagnosed celiac disease cases was 4.7 years (SD 4.0) and the median age was 2.75 years; they had thus been on a gluten-free diet for approximately 9 years since the screening was performed at 12 years of age. Adherence to a gluten-free diet among children is generally good [26–28]. In our cohort, 83 out of the 93 previously diagnosed cases had normal tTG at the time of the screening as a measure of very good compliance and only 10 out of 93 had slightly raised tTG (range 4.1 to 29.95) above normal. According to our study results, a gluten-free diet does not seem to be protective against the development of thyroid autoimmunity.

The results are in accordance with those of Sategna Guidetti et al. who observed that many celiac patients developed autoimmune disorders despite strict adherence to a gluten-free diet [29]. Viljamaa et al. also investigated the prevalence of autoimmune disease associated with celiac disease among both adults and children. They found that the duration of gluten exposure did not seem to be of crucial importance regarding development of autoimmune diseases. One-third of the patients in their study developed associated autoimmune diseases despite being on a gluten-free diet [16]. In fact, in the latter study the development of autoimmune disease decreased with duration of gluten exposure. One can hypothesize that these patients, who were diagnosed with celiac disease at a later age, might be generally less susceptible to developing autoimmune diseases. This is also supported by Cosnes et al. who showed that a late diagnosis of celiac disease was associated with a decreased risk of autoimmunity [26]. The above-mentioned findings might support a common genetic susceptibility to the autoimmune conditions. It is important to bear in mind that autoimmune diseases develop at different ages; for example, type 1 diabetes mellitus often develops during childhood, while development of autoimmune thyroiditis increases with increasing age and has its peak incidence in the fifth decade of life [30, 31]. It is thus important to analyze the incidence of autoimmune diseases in relation to both age and compliance with a gluten-free diet. Cosnes et al. found that the risk of development of an autoimmune disease in celiac patients was increased in patients with a family history of autoimmune disease when celiac disease was diagnosed early in childhood or adolescence compared to adulthood [26]. The fact that family history was a strong contributing factor to the development of autoimmune disease also favors a linkage disequilibrium between the genes responsible for celiac disease and those responsible for the coexpressed autoimmune disease [32].

Contrary to the abovementioned findings, Ventura et al. showed that patients with celiac disease had a high prevalence of both insulin dependent diabetes mellitus autoantibodies and thyroid-related serum autoantibodies. These autoantibodies were supposed to be gluten-dependent, since they disappeared during treatment with a gluten-free diet [12]. They also observed that the prevalence of autoimmune disorders in children in whom celiac disease was diagnosed before the age of two years, and who were treated with a gluten-free diet, was comparable to that of controls [18]. In our study, the distribution of TPO positivity at 12 years of age among the previously diagnosed celiac disease cases was equal among those diagnosed before 2 years of age and after 2 years of age. It was also equally distributed regardless of the degree of mucosal damage in the entire celiac disease cohort.

Sategna Guidetti et al. hypothesized that gluten ingestion plays a central role in modifying the immunological response early in life [29]. As mentioned above, we could not verify this theory in our study since we did not find any difference in the prevalence of TPOAb among the screening-detected, and thus untreated, celiac disease cases and the previously diagnosed celiac disease cases. However, some of the previously diagnosed cases in our study were diagnosed at a later age than before the age of two. If the hypothesis of Sategna Guidetti et al. is true and this is a crucial age, the opportunity to modify the immunological response might have disappeared after that age.

The present trial was based on a nationwide, contemporary study on celiac disease in 12632 children/almost 13000 children. Yet, it has some limitations that merit consideration. First, celiac disease was defined pathologically as Marsh 1–3c. The inclusion of Marsh 1 in the diagnosis is currently an unsettled issue in the scientific community. We chose to include Marsh 1 in accordance with previous publications [3, 19] and research showing that patients with low-grade inflammation still benefit from a gluten-free diet [33]. Second, albeit the screening study included 12632 children, power nevertheless constitutes a limitation of the current study, as diagnosis of concomitant celiac disease and elevated TPOAb was relatively rare in our cohorts. Thus, the lack of a significant difference in TPOAb between the screening-detected and previously detected CD cases could possibly be due to a lack of power. The third limitation of the present study is that we only analyzed TPO markers as an indicator of autoimmune disease. We did not investigate how many of the children developed clinical autoimmune thyroiditis, defined as TPOAb positivity in combination with goiter and/or hypothyroidism. Although the clinical significance of these antibodies in celiac disease is still unclear, there is probably a higher propensity for thyroid autoimmunity in children with positive TPO markers. In most cases the immune response to the target cells progressively destroys the endocrine gland, and hypofunction is the main clinical manifestation [34]. The presence of TPOAb in serum is an independent risk factor for the development of hypothyroidism in patients with subclinical hypothyroidism [35, 36]. The Whickham study demonstrated that the presence of antithyroid microsomal (TPO is the antigen involved in the “microsomal” response) antibodies, or elevated serum TSH alone, was associated with a significant increased risk of developing hypothyroidism at 20 years of age [36]. A longitudinal followup would, therefore, seem necessary in patients with positive autoimmune thyroid serology but who are currently euthyroid.

## 5. Conclusion

Thus far, we can conclude that having celiac disease as a 12-year old increases the risk of also having thyroid autoimmunity almost threefold compared to healthy children. Our findings do not support a general screening for celiac disease on the basis of trying to protect against thyroid disease through earlier diagnosis of celiac disease and initiation of treatment with a gluten-free diet.

## Figures and Tables

**Table 1:** Risk for thyroid peroxidase antibody (TPO) positivity in 12-year olds when comparing treated and untreated celiac disease (CD) cases with non-CD children through a case-referent study nested within the ETICS^1^ study.

Groups	TPO^2^				OR^3^	95% CI^3^
	Positive (*n* = 72)		Negative (*n* = 1958)			
	*n*	%^4^	*n*	%		
Non-CD^5^	48	2.8	1647	97.2	1.0	—
Previously diagnosed CD	7	7.5	86	92.5	2.8	1.2–6.4
Screening-detected CD	17	7.0	225	93.0	2.6	1.5–4.6

^1^Exploring the Iceberg of celiacs in Sweden (ETICS).

^2^The thyroid peroxidase (TPO) cut-off used was 100 U/mL.

^3^Logistic regression was used to estimate odds ratio (OR) with 95% confidence interval (CI).

^4^Row percentages.

^5^Children not diagnosed with celiac disease (CD).

## Abbreviations

tTG: Transglutaminase
EMA: Endomysial antibodies
TPOAb: Thyroid peroxidase antibodies
OR: Odds ratio
SD: Standard deviation
CI: Confidence interval.
